# Supplementary figures and images for: A fairer way to compare researchers at any career stage and in any discipline using open-access citation data
Source: PLoS One. 2021 Sep 10;16(9):e0257141. doi: 10.1371/journal.pone.0257141 (PMC8432834; doi:10.1371/journal.pone.0257141)

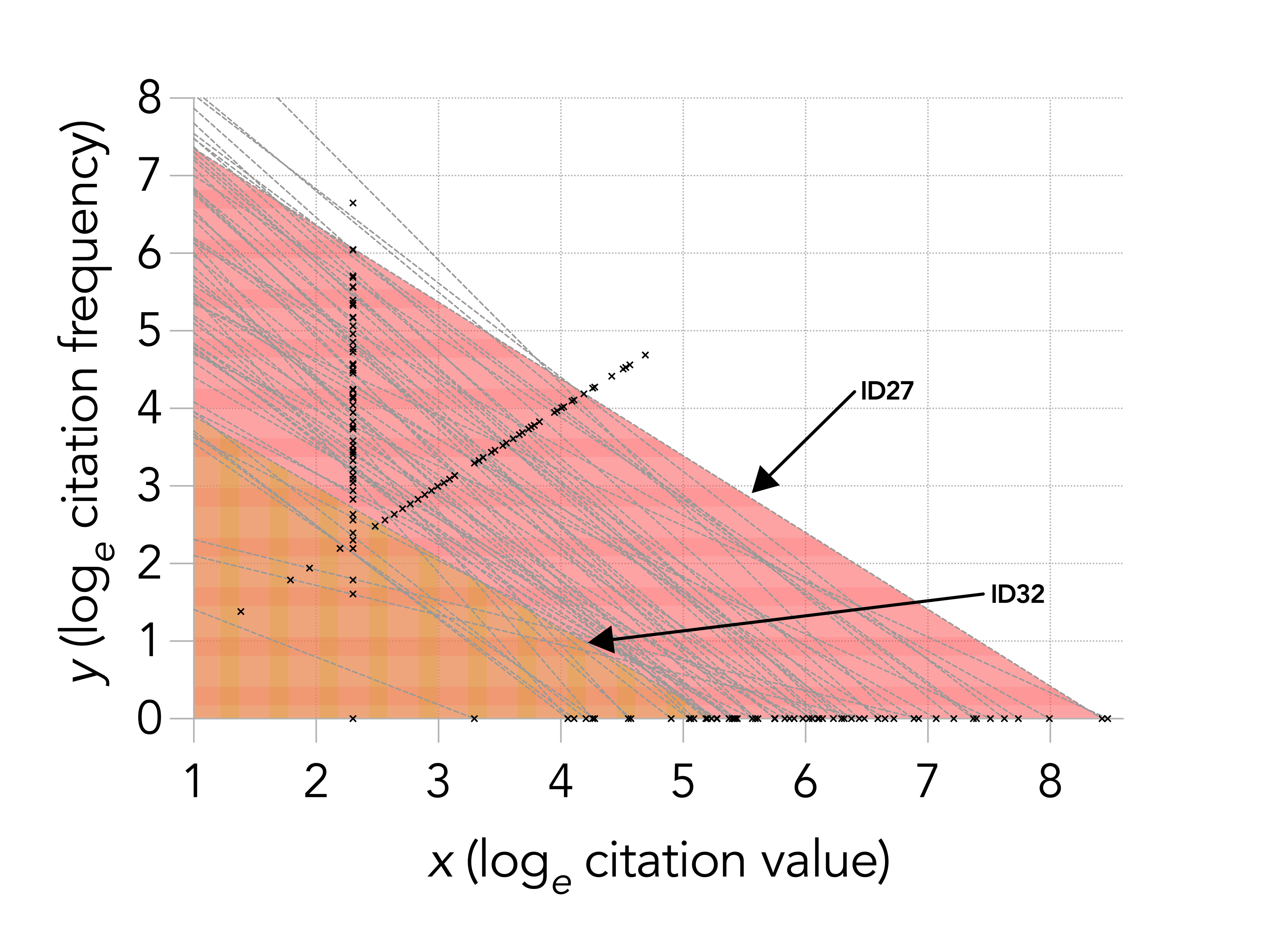

Supplement: S1 Fig — Relationship between loge citation frequency (y) and loge citation value (x) for 60 researchers within the discipline of ophthalmology. Each light grey, dashed line is the linear (on the loge-loge scale) fit for each individual researcher. The area under the fitted line (Arel) is shown for individual 32 (ID32; red horizontal hatch) and individual 27 (orange vertical hatch). (TIF) [file pone.0257141.s001.tif]

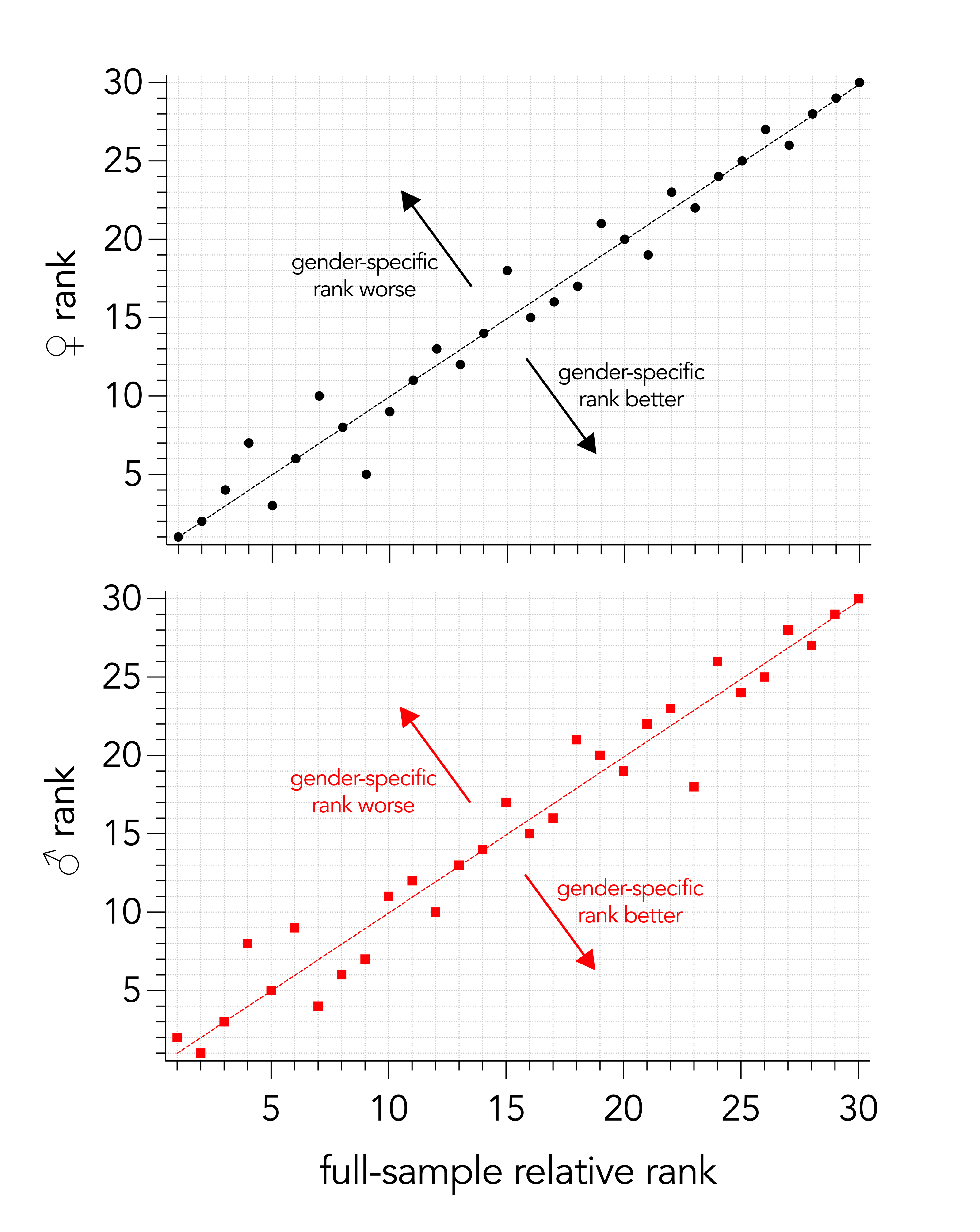

Supplement: S3 Fig — Gender-specific researcher ranks versus ranks derived from the entire sample (in this case, the microbiology sample shown in S2 Fig). For women who increased ranks when only compared to other women (negative residuals; top panel), the average increase was 1.50 places higher. For women with reduced ranks (positive residuals; top panel), the average was 1.88 places lower. For men who increased ranks when only compared to other men (negative residuals; bottom panel), or who declined in rank (positive residuals; bottom panel), the average number of places moved were both 1.75 for both. (TIF) [file pone.0257141.s003.tif]

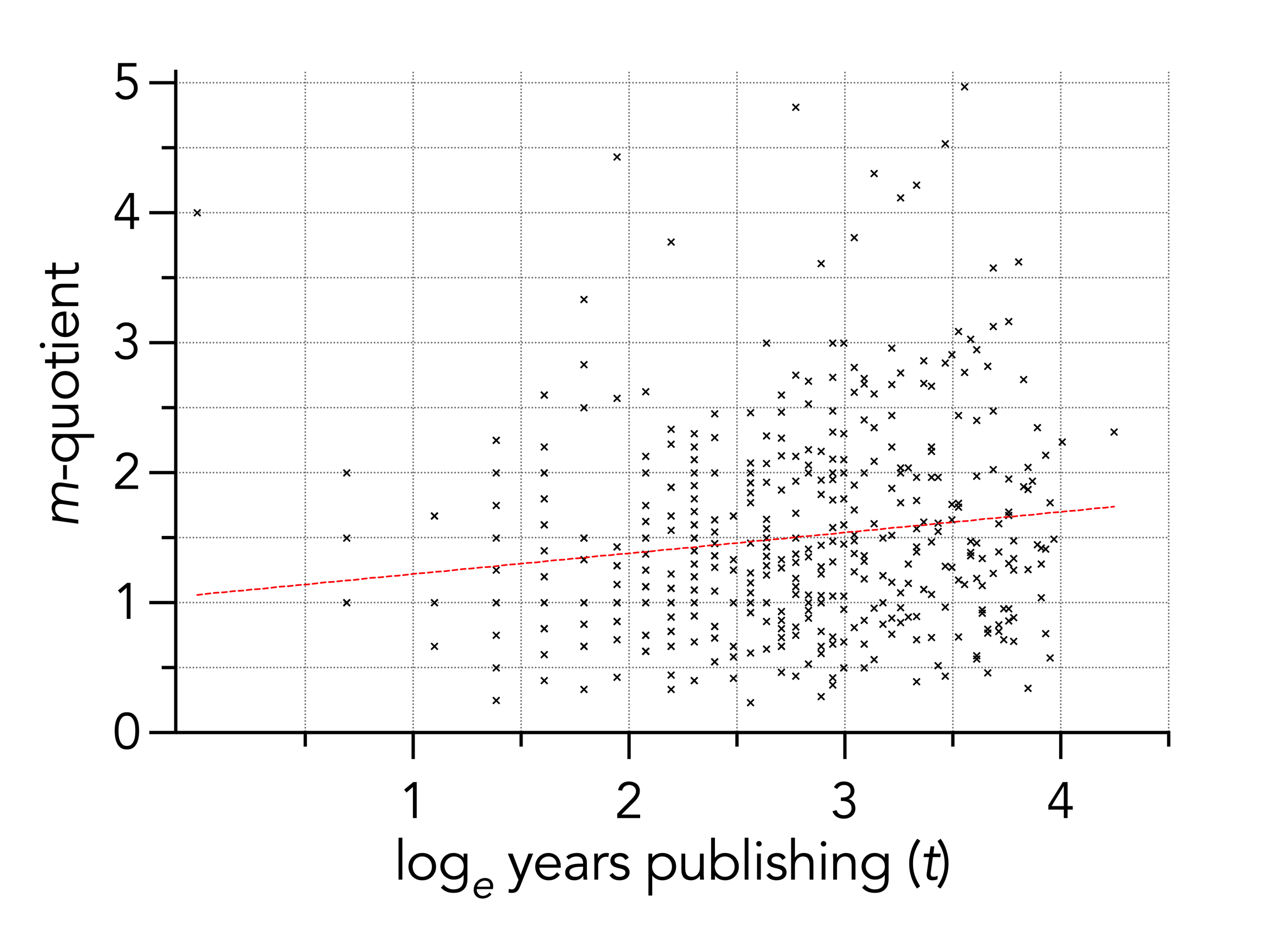

Supplement: S4 Fig — Relationship between the m-quotient and loge years publishing (t) for 480 researchers in eight different disciplines. There is a weak, but statistically supported positive relationship (information-theoretic evidence ratio = 68.7). (TIF) [file pone.0257141.s004.tif]

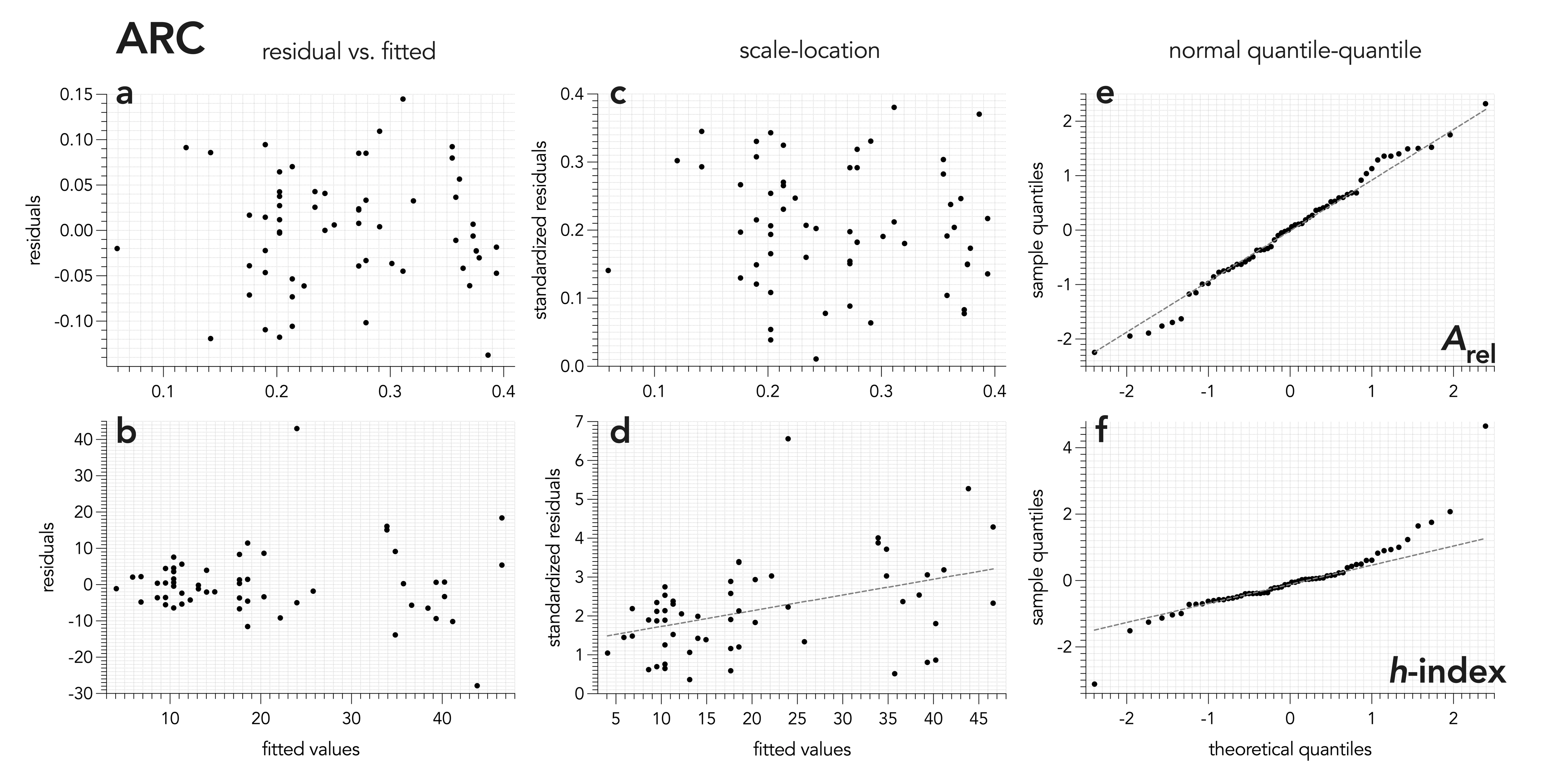

Supplement: S5 Fig — Residual vs. fitted (a & b), scale-location (c & d), and normal quantile-quantile (e & f) plots for the relationship between loge Arel (area under the power-law relationship) and loge t (years publishing) used to derive the ε-index (top row), and for the relationship between the h-index and t used to derive the m-quotient (bottom row) for 60 researchers in the discipline of archaeology (ARC). The Arel ~ loge(t) relationships show homoscedasticity (i.e., a random pattern in the residual vs. fitted plots, and no trend in the scale-location plots) and a near-Normal distribution (points fall on the expected quantile-quantile line). In contrast, the h-index ~ t relationships all show heteroscedasticity (i.e., a ‘fan’ pattern in the residual vs. fitted plots, and a positive trend in the scale-location plots) and a non-Normal distribution (points diverge considerably more from the expected quantile-quantile line). (TIF) [file pone.0257141.s005.tif]

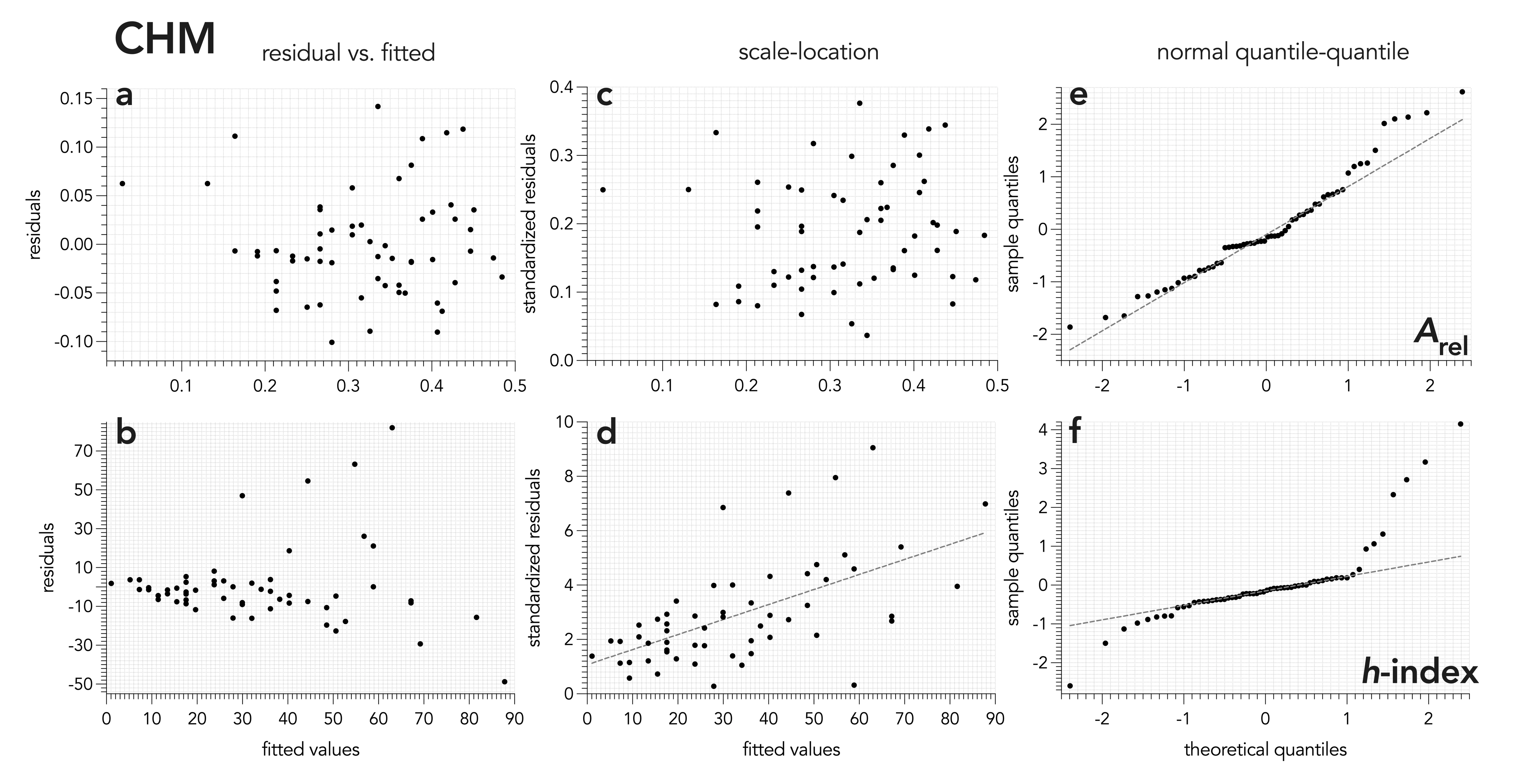

Supplement: S6 Fig — Residual vs. fitted (a & b), scale-location (c & d), and normal quantile-quantile (e & f) plots for the relationship between loge Arel (area under the power-law relationship) and loge t (years publishing) used to derive the ε-index (top row), and for the relationship between the h-index and t used to derive the m-quotient (bottom row) for 60 researchers in the discipline of chemistry (CHM). The Arel ~ loge(t) relationships show homoscedasticity (i.e., a random pattern in the residual vs. fitted plots, and no trend in the scale-location plots) and a near-Normal distribution (points fall on the expected quantile-quantile line). In contrast, the h-index ~ t relationships all show heteroscedasticity (i.e., a ‘fan’ pattern in the residual vs. fitted plots, and a positive trend in the scale-location plots) and a non-Normal distribution (points diverge considerably more from the expected quantile-quantile line). (TIF) [file pone.0257141.s006.tif]

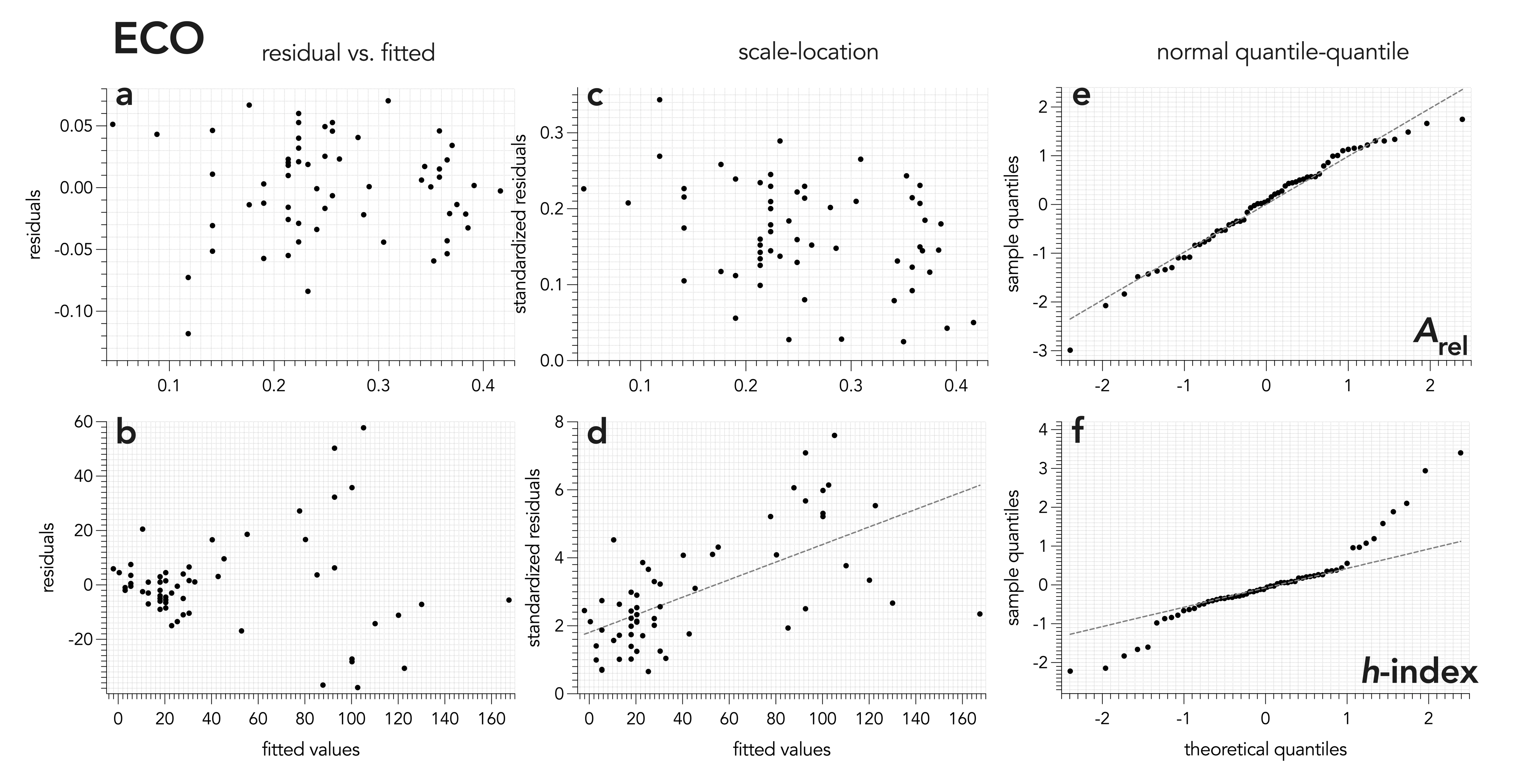

Supplement: S7 Fig — Residual vs. fitted (a & b), scale-location (c & d), and normal quantile-quantile (e & f) plots for the relationship between loge Arel (area under the power-law relationship) and loge t (years publishing) used to derive the ε-index (top row), and for the relationship between the h-index and t used to derive the m-quotient (bottom row) for 60 researchers in the discipline of ecology (ECO). The Arel ~ loge(t) relationships show homoscedasticity (i.e., a random pattern in the residual vs. fitted plots, and no trend in the scale-location plots) and a near-Normal distribution (points fall on the expected quantile-quantile line). In contrast, the h-index ~ t relationships all show heteroscedasticity (i.e., a ‘fan’ pattern in the residual vs. fitted plots, and a positive trend in the scale-location plots) and a non-Normal distribution (points diverge considerably more from the expected quantile-quantile line). (TIF) [file pone.0257141.s007.tif]

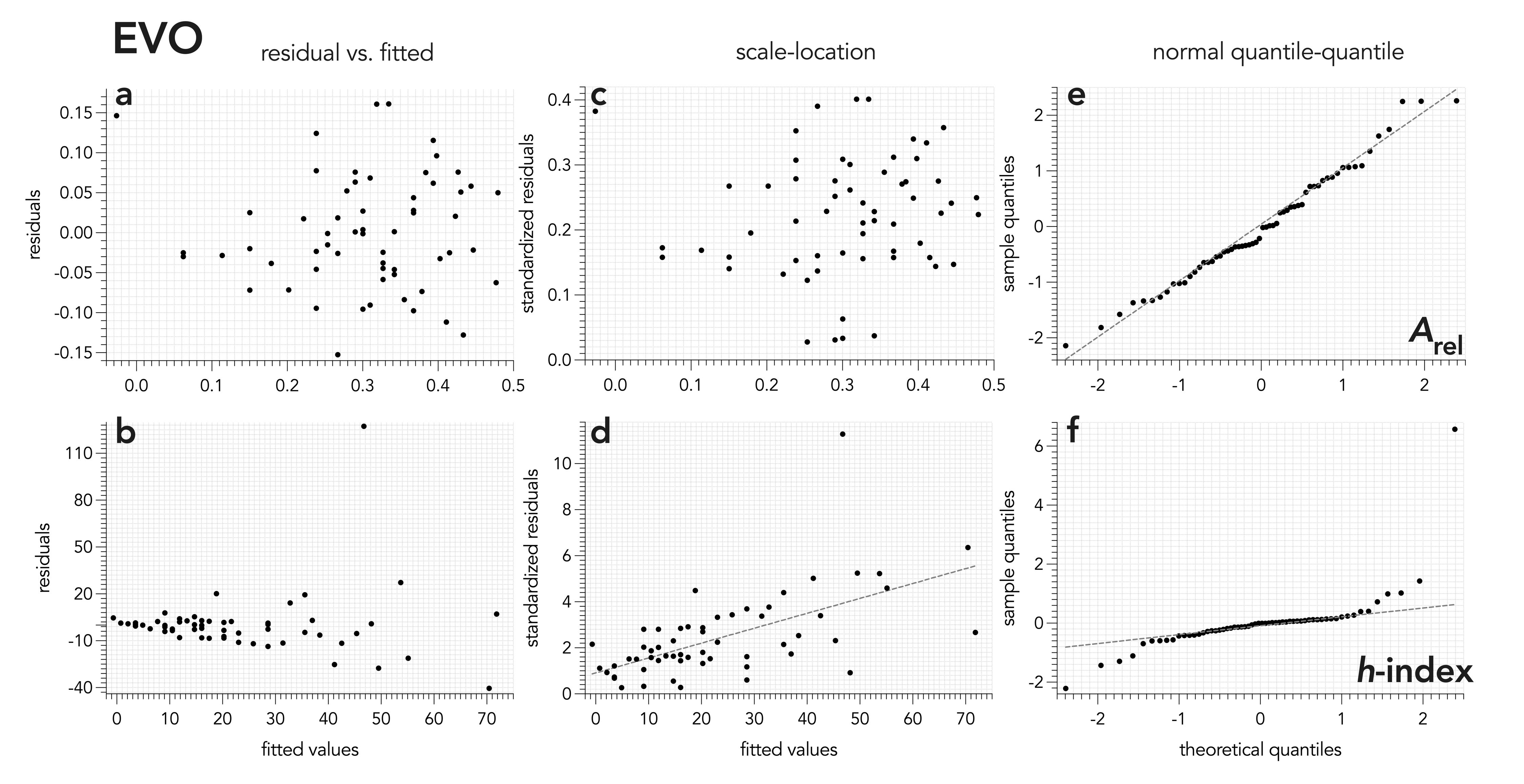

Supplement: S8 Fig — Residual vs. fitted (a & b), scale-location (c & d), and normal quantile-quantile (e & f) plots for the relationship between loge Arel (area under the power-law relationship) and loge t (years publishing) used to derive the ε-index (top row), and for the relationship between the h-index and t used to derive the m-quotient (bottom row) for 60 researchers in the discipline of evolution and development (EVO). The Arel ~ loge(t) relationships show homoscedasticity (i.e., a random pattern in the residual vs. fitted plots, and no trend in the scale-location plots) and a near-Normal distribution (points fall on the expected quantile-quantile line). In contrast, the h-index ~ t relationships all show heteroscedasticity (i.e., a ‘fan’ pattern in the residual vs. fitted plots, and a positive trend in the scale-location plots) and a non-Normal distribution (points diverge considerably more from the expected quantile-quantile line). (TIF) [file pone.0257141.s008.tif]

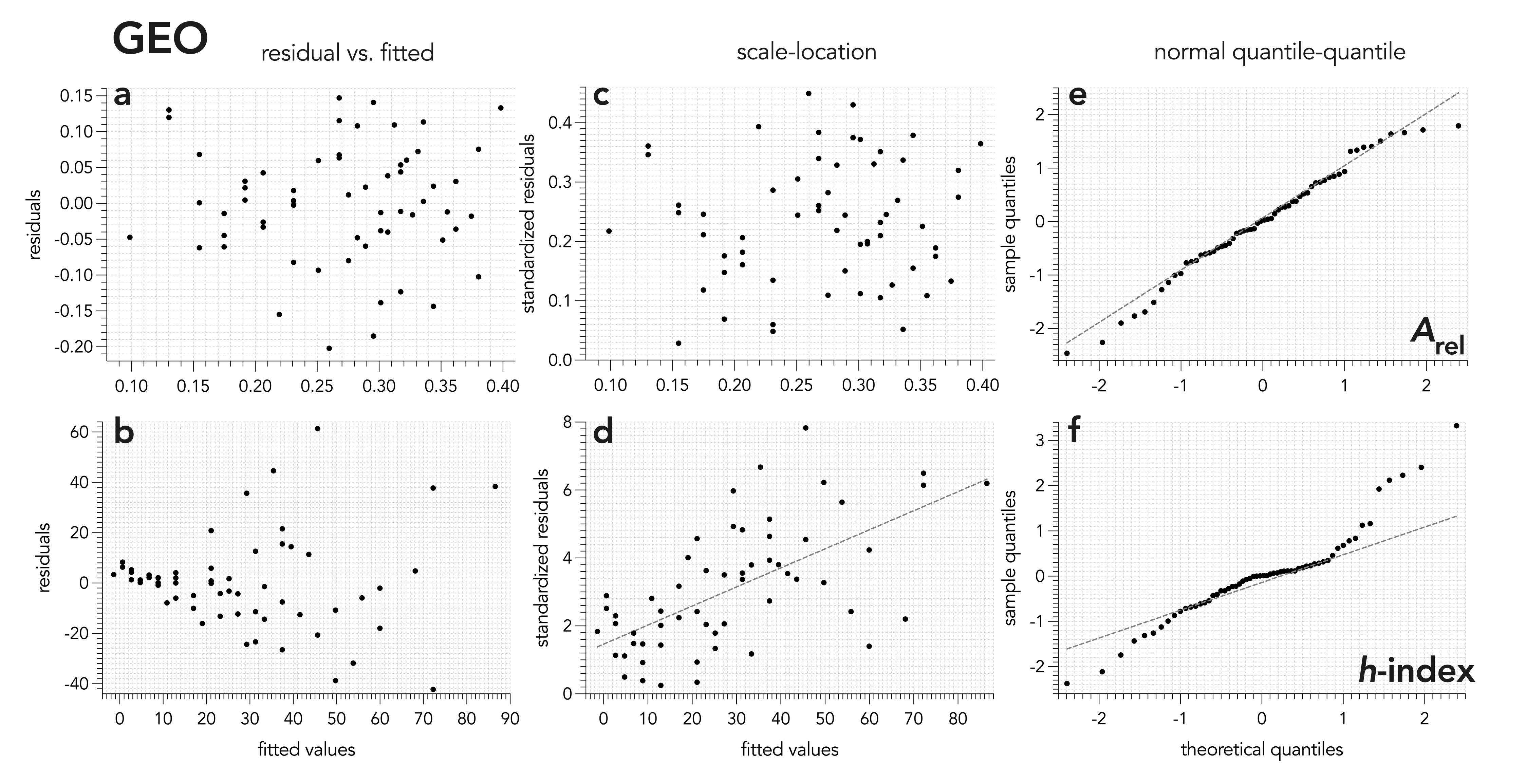

Supplement: S9 Fig — Residual vs. fitted (a & b), scale-location (c & d), and normal quantile-quantile (e & f) plots for the relationship between loge Arel (area under the power-law relationship) and loge t (years publishing) used to derive the ε-index (top row), and for the relationship between the h-index and t used to derive the m-quotient (bottom row) for 60 researchers in the discipline of geology (GEO). The Arel ~ loge(t) relationships show homoscedasticity (i.e., a random pattern in the residual vs. fitted plots, and no trend in the scale-location plots) and a near-Normal distribution (points fall on the expected quantile-quantile line). In contrast, the h-index ~ t relationships all show heteroscedasticity (i.e., a ‘fan’ pattern in the residual vs. fitted plots, and a positive trend in the scale-location plots) and a non-Normal distribution (points diverge considerably more from the expected quantile-quantile line). (TIF) [file pone.0257141.s009.tif]

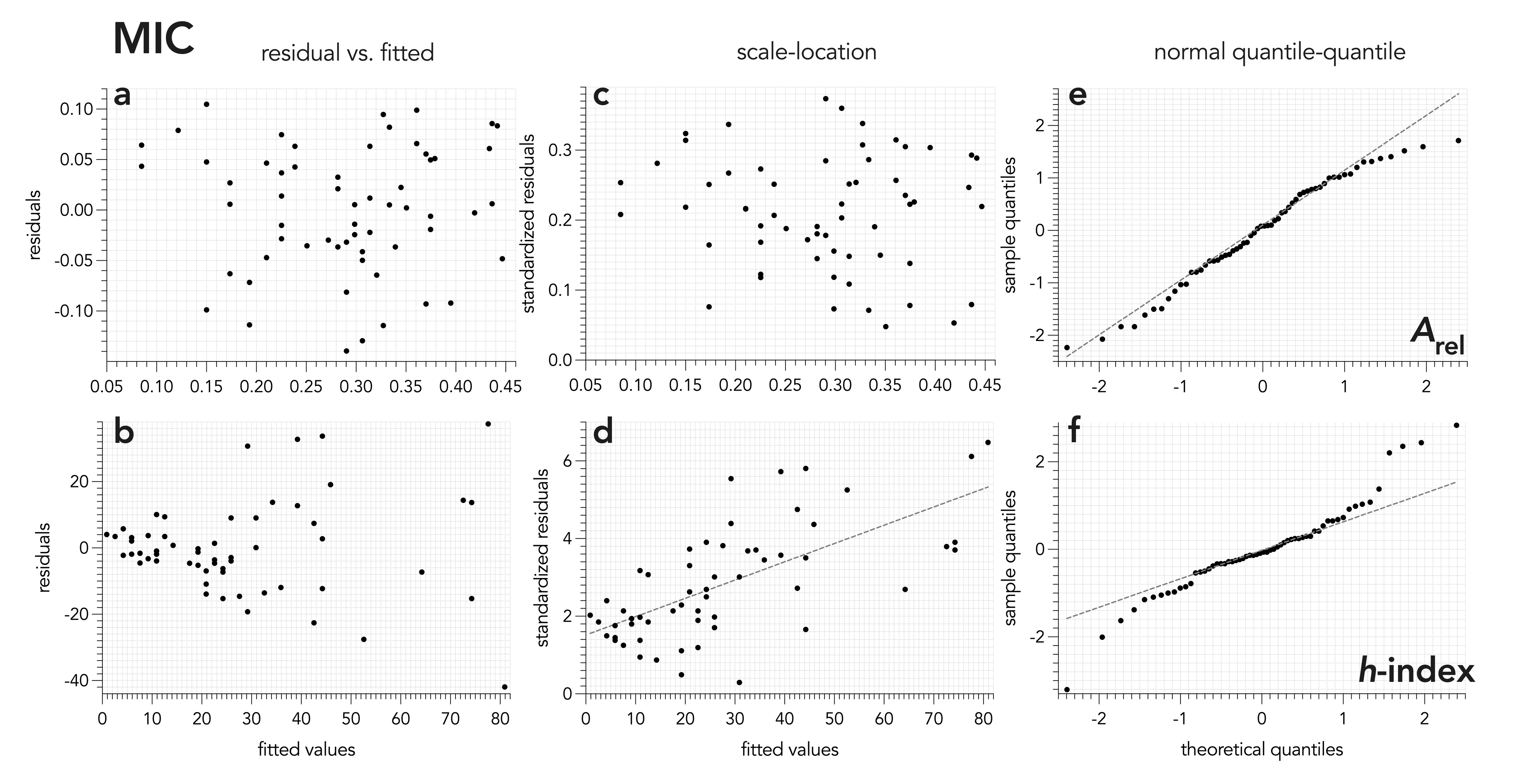

Supplement: S10 Fig — Residual vs. fitted (a & b), scale-location (c & d), and normal quantile-quantile (e & f) plots for the relationship between loge Arel (area under the power-law relationship) and loge t (years publishing) used to derive the ε-index (top row), and for the relationship between the h-index and t used to derive the m-quotient (bottom row) for 60 researchers in the discipline of microbiology (MIC). The Arel ~ loge(t) relationships show homoscedasticity (i.e., a random pattern in the residual vs. fitted plots, and no trend in the scale-location plots) and a near-Normal distribution (points fall on the expected quantile-quantile line). In contrast, the h-index ~ t relationships all show heteroscedasticity (i.e., a ‘fan’ pattern in the residual vs. fitted plots, and a positive trend in the scale-location plots) and a non-Normal distribution (points diverge considerably more from the expected quantile-quantile line). (TIF) [file pone.0257141.s010.tif]

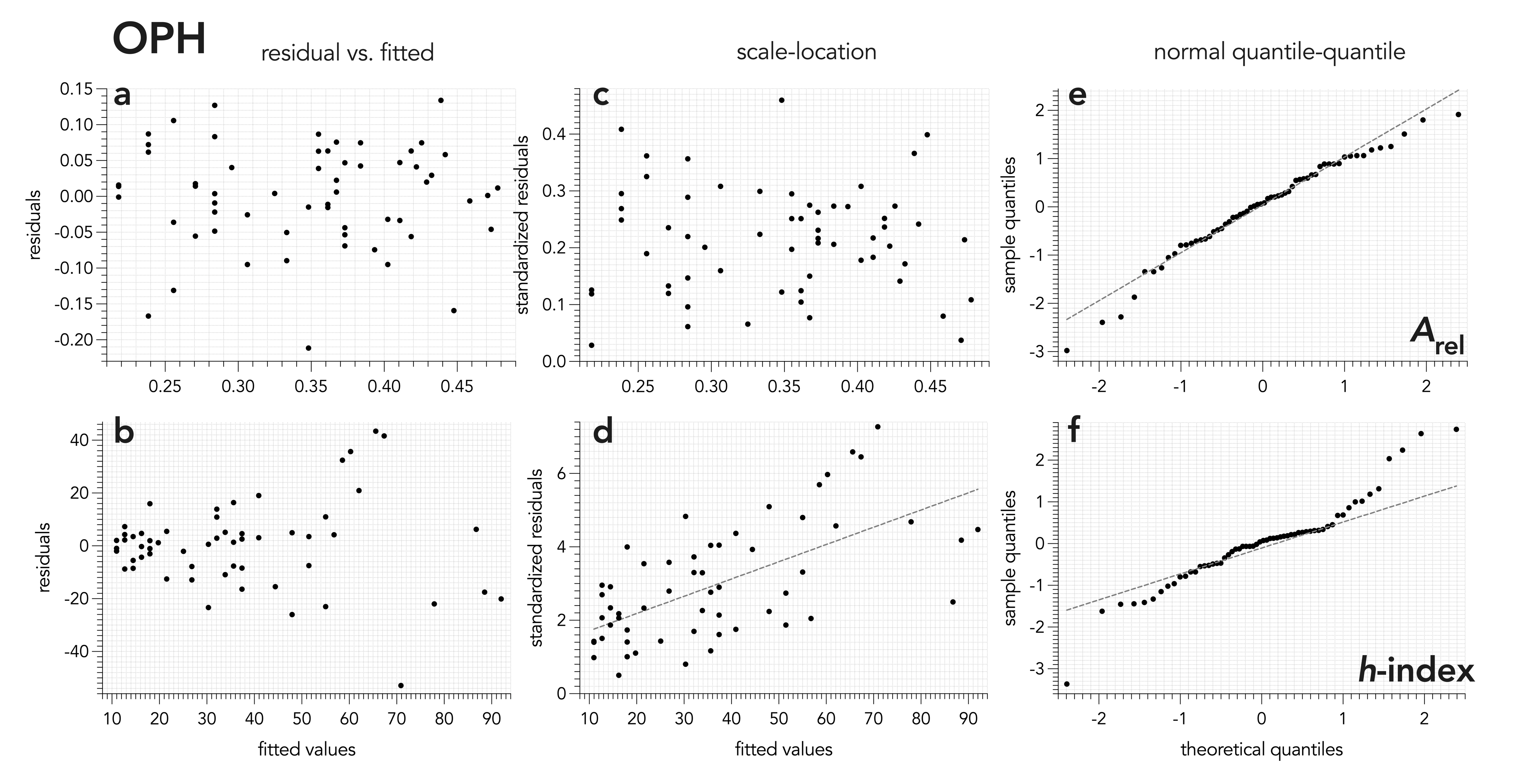

Supplement: S11 Fig — Residual vs. fitted (a & b), scale-location (c & d), and normal quantile-quantile (e & f) plots for the relationship between loge Arel (area under the power-law relationship) and loge t (years publishing) used to derive the ε-index (top row), and for the relationship between the h-index and t used to derive the m-quotient (bottom row) for 60 researchers in the discipline of ophthalmology (OPH). The Arel ~ loge(t) relationships show homoscedasticity (i.e., a random pattern in the residual vs. fitted plots, and no trend in the scale-location plots) and a near-Normal distribution (points fall on the expected quantile-quantile line). In contrast, the h-index ~ t relationships all show heteroscedasticity (i.e., a ‘fan’ pattern in the residual vs. fitted plots, and a positive trend in the scale-location plots) and a non-Normal distribution (points diverge considerably more from the expected quantile-quantile line). (TIF) [file pone.0257141.s011.tif]

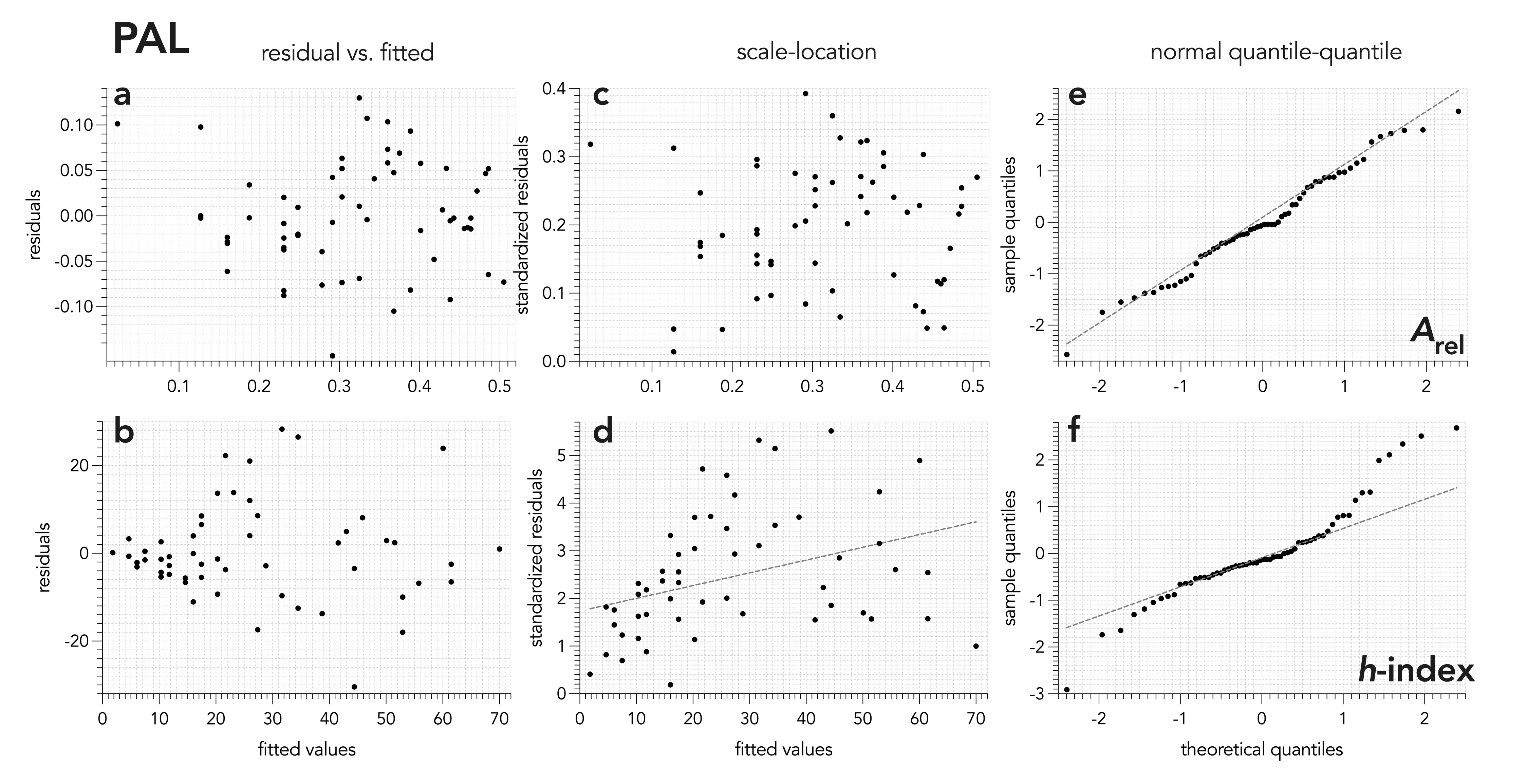

Supplement: S12 Fig — Residual vs. fitted (a & b), scale-location (c & d), and normal quantile-quantile (e & f) plots for the relationship between loge Arel (area under the power-law relationship) and loge t (years publishing) used to derive the ε-index (top row), and for the relationship between the h-index and t used to derive the m-quotient (bottom row) for 60 researchers in the discipline of palaeontology (PAL). The Arel ~ loge(t) relationships show homoscedasticity (i.e., a random pattern in the residual vs. fitted plots, and no trend in the scale-location plots) and a near-Normal distribution (points fall on the expected quantile-quantile line). In contrast, the h-index ~ t relationships all show heteroscedasticity (i.e., a ‘fan’ pattern in the residual vs. fitted plots, and a positive trend in the scale-location plots) and a non-Normal distribution (points diverge considerably more from the expected quantile-quantile line). (TIF) [file pone.0257141.s012.tif]
